# Effectiveness of acid suppressants and other mucoprotective agents in reducing the risk of occult gastrointestinal bleeding in nonsteroidal anti-inflammatory drug users

**DOI:** 10.1038/s41598-019-48173-6

**Published:** 2019-08-12

**Authors:** Tae Jun Kim, Eun Ran Kim, Sung Noh Hong, Young-Ho Kim, Yeong Chan Lee, Hye Seung Kim, Kyunga Kim, Dong Kyung Chang

**Affiliations:** 1 Department of Medicine, Samsung Medical Center, Sungkyunkwan University School of Medicine, Seoul, South Korea; 2 Department of Digital Health, Samsung Advanced Institute for Health Science and Technology, Sungkyunkwan University School of Medicine, Seoul, South Korea; 3 Biostatistics and Clinical Epidemiology Center, Samsung Medical Center, Sungkyunkwan University School of Medicine, Seoul, South Korea

**Keywords:** Gastrointestinal bleeding, Lower gastrointestinal bleeding, Upper gastrointestinal bleeding

## Abstract

Acid suppressants such as histamine-2 receptor antagonists (H2RAs) and proton pump inhibitors (PPIs) are effective in preventing gastrointestinal (GI) bleeding in nonsteroidal anti-inflammatory drugs (NSAIDs) users. Despite widespread acid suppressant use, there remain concerns about several potential risks of long-term use. Therefore, we investigated whether gastroprotective agents (GPAs) other than acid suppression therapy are effective in preventing NSAID-related GI injury. To this end, we studied 9,133 patients with osteoarthritis or rheumatoid arthritis who used NSAIDs for ≥1 month. A decrease of 2 g/dL or more in the hemoglobin level was considered a GI injury indicator. The GPAs included acid suppressants and other mucoprotective agents. Acid suppressants included PPIs and H2RAs. Other mucoprotective agents included misoprostol, rebamipide, and eupatilin. During a median follow-up period of 27 (range, 4.3-51.3) weeks, occult GI bleeding occurred in 1,191 (13%) patients. A comparison of patients who used GPAs concomitantly with that of nonusers in a multivariable analysis revealed the hazard ratios (HRs; 95% confidence intervals [CIs]) for occult GI bleeding were 0.30 (0.20-0.44), 0.35 (0.29-0.43), 0.47 (0.23-0.95), 0.43 (0.35-0.51), and 0.98 (0.86-1.12) for PPIs, H2RAs, misoprostol, rebamipide, and eupatilin, respectively. Compared to PPI co-treatment, H2RA, misoprostol, rebamipide, and eupatilin co-treatments were associated with occult GI bleeding HRs (95% CIs) of 1.19 (0.79-1.79), 1.58 (0.72-3.46), 1.44 (0.96-2.16), and 3.25 (2.21-4.77), respectively. Our findings suggest that mucoprotective agents, such as rebamipide and misoprostol, as well as acid suppressants, are effective in reducing the risk for GI injury in NSAID users.

## Introduction

Nonsteroidal anti-inflammatory drugs (NSAIDs) are one of the most widely prescribed drugs worldwide because of their analgesic, anti-inflammatory, and antipyretic effects. However, despite the various benefits and good efficacy of NSAIDs, their use can lead to mucosal injury of the gastrointestinal (GI) tract including GI bleeding or perforation. The incidence of clinically significant upper GI bleeding in NSAID users has been estimated at 1-2.5/100 person-years^1,2^. Current evidence suggests that in addition to causing upper GI bleeding, NSAIDs increase the risk of lower GI bleeding to a similar extent as that of upper GI bleeding^3–5^.

Substantial evidence has indicated that the concomitant use of gastroprotective agents (GPAs) in chronic NSAID users can reduce the risk of upper GI bleeding^6,7^. Thus, many practice guidelines recommend using GPAs in NSAID users with a high risk of GI bleeding^8–10^. However, the treatment and prevention of NSAID-associated lower GI bleeding remain unclear and difficult, because the pathogenic mechanisms are different and not well understood. GPAs mainly include acid suppressants, such as proton pump inhibitors (PPIs) and histamine-2 receptor antagonists (H2RAs), which primarily have a protective effect against upper GI damage. GPAs other than acid suppressants, including misoprostol, rebamipide, and eupatilin, have a different GI tract–protective mechanism. Further, in some studies, misoprostol and rebamipide were reported to be effective against NSAID-associated enteropathy; however, the underlying mechanisms remain unclear^11–13^.

A decrease of ≥2 g/dL in the hemoglobin level has been suggested as a clinically-relevant indicator of GI mucosal damage and has previously been evaluated as an endpoint of GI bleeding in several studies^14–18^. Thus, a ≥ 2 g/dL drop in the hemoglobin level could be a potential marker for NSAID-induced mucosal damage throughout the GI tract, including both upper and lower GI events.

Recently, a prospective study of 84 aspirin users showed that misoprostol was superior to placebo in treating small bowel ulcers complicated by small bowel ulcer bleeding in aspirin users^19^. Another prospective study of 104 patients who were taking low-dose aspirin or an NSAID, demonstrated that misoprostol was effective for the treatment of small bowel ulcers and erosions^20^. Further, two prospective studies of 80 patients and 20 patients who were taking NSAIDs reported that rebamipide showed significantly higher efficacy than that of placebo in preventing NSAID-induced small bowel mucosal injury^11,21^. However, to the best of our knowledge, no large-scale study has evaluated the risk for GI bleeding in NSAID users with the concomitant use of a GPA, especially rebamipide or misoprostol. Therefore, we conducted a large cohort study to determine the risk of overall GI bleeding in NSAID users by using different GPA co-treatments. Furthermore, we investigated whether GPAs other than PPIs or H2RAs are effective in preventing NSAID-associated occult GI bleeding.

## Results

A total of 6,465 GPA plus NSAID users and 2,668 NSAID users without GPA were included in this study. The most frequent GPA used was eupatilin (29.8%), followed by H2RAs (18.1%), rebamipide (16.5%), PPI (5.5%), and misoprostol (0.9%). The mean (standard deviation) age of the 9,133 study participants was 54.2 (14.7) years. The baseline characteristics of the participants according to concomitant GPA use are shown in Table 1. The proportions of male and female patients were comparable in both groups. Patients without GPA co-treatment were more likely to be older, and to have diabetes and lower levels of creatinine compared to those with GPA co-treatment. The proportions of comorbidities such as hypertension, cerebrovascular disease, and ischemic heart disease were comparable between both groups.

**Table 1:** Baseline characteristics of study participants according to GPA use.

	Total (N = 9,133)	Monotherapy of NSAID (N = 2,668)	NSAID plus GPA (N = 6,465)	*P* value
Age, years	54.2 ± 14.7	55.7 ± 13.6	53.7 ± 15	<0.001
Male	3,145 (34.4)	915 (34.3)	2,230 (34.5)	0.879
BMI (kg/m^2^)	26.8 ± 4.7	27.7 ± 4.6	26.4 ± 4.5	0.121
Creatinine (mg/dl)	0.7 ± 0.46	0.7 ± 0.5	0.8 ± 0.4	<0.001
**Co-morbidity**				
Hypertension	1,133 (12.4)	325 (12.2)	808 (12.5)	0.456
Diabetes mellitus	273 (3)	99 (3.7)	174 (2.7)	0.011
Cerebrovascular disease	103 (1.1)	32 (1.2)	71 (1.1)	0.759
Ischemic heart disease	246 (2.7)	64 (2.4)	182 (2.8)	0.295
**Concomitant GPAs**				
None	2,668 (29.2)	2,668 (100)	0	
PPI	498 (5.5)	0	498 (7.7)	
H2RA	1,654 (18.1)	0	1,654 (25.6)	
Misoprostol	85 (0.9)	0	85 (1.3)	
Rebamipide	1,509 (16.5)	0	1,509 (23.3)	
Eupatilin	2,719 (29.8)	0	2,719 (42.1)	

Values are expressed as means ± standard deviation or percentages.

**Figure 1:**
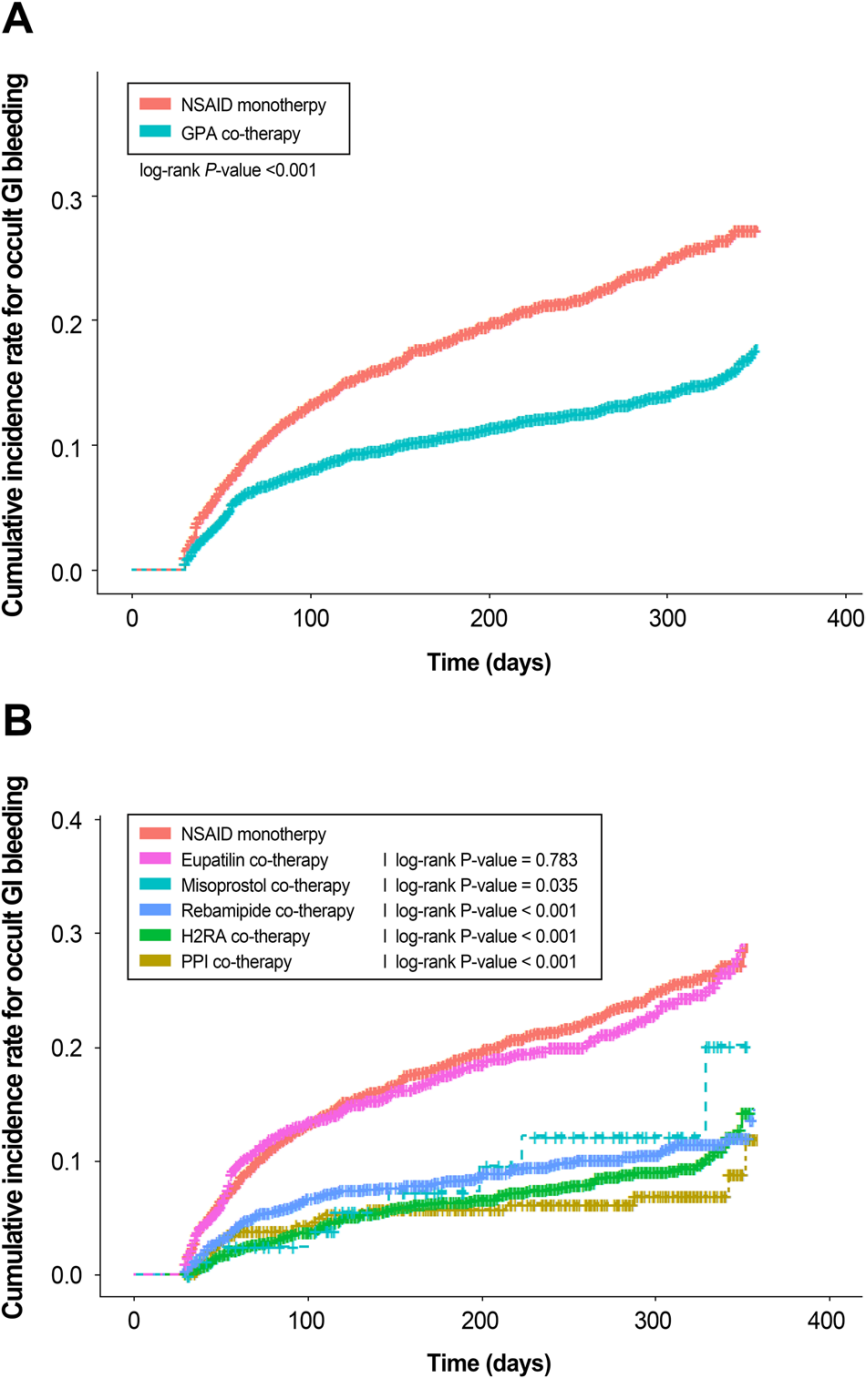
(**A**) Cumulative incidence rate for occult gastrointestinal bleeding between NSAID monotherapy and GPA co-therapy, (**B**) Cumulative incidence rate for occult gastrointestinal bleeding according to each drug combinations.

During the median follow-up of 5.7 months (interquartile range, 2.0-9.8 months), occult GI bleeding developed in 1,191 (13%) patients. Of the 1,191 patients with occult bleeding, 82 had endoscopically-confirmed GI bleeding. Sixty-five patients had upper GI bleeding and 17 had lower GI bleeding. Among those with upper GI bleeding, the most cause of the bleeding was peptic ulcer (54 cases). Lower GI bleeding was noted in seven patients with angiodysplasia, six patients with ulcers, and four patients with diverticular bleeding. Figure 1A shows the Kaplan-Meier curves of the proportion of patients who developed occult GI bleeding during the 12-month study, according to concomitant GPA use. The incident cases (%) of occult GI bleeding in each group were 703 (10.9%) in the GPA co-treatment group and 488 (18.3%) in the no-GPA co-treatment group (*P* < 0.001). The comparison of concomitant GPA users with nonusers by using the multivariable Cox proportional hazard model, adjusted for age, sex, body mass index (BMI), creatinine level, and comorbidities, showed the HR (95% CI) for occult GI bleeding was 0.63 (0.56-0.71, *P* < 0.001). Furthermore, the multivariable analysis showed that increasing age and a history of ischemic heart disease were significant risk factors for occult GI bleeding (Table 2).

**Table 2:** The risk of GI bleeding in multivariable analysis.

	Multivariable analysis		
	HR	95% CI	*P* value
Age, years	1.04	1.03-1.05	<0.001
Male	0.89	0.79-1.02	0.095
BMI (kg/m^2^)	1.00	1.00-1.01	0.067
Creatinine (mg/dl)	1.01	0.88-1.16	0.877
**Co-morbidity**			
Hypertension	1.04	0.87-1.24	0.634
Diabetes mellitus	1.12	0.83-1.51	0.454
Cerebrovascular disease	1.90	1.22-2.97	0.005
Ischemic heart disease	1.61	1.20-2.14	0.001
Concomitant any GPAs use	0.63	0.56-0.71	<0.001

The cumulative incidences of occult GI bleeding based on GPA combinations during the 12-month study are shown in Fig. 1B. During the median follow-up of 6 months (interquartile range, 1.9-10 month), the incident cases (%) of occult GI bleeding were 28 (5.6%) for PPIs, 133 (8%) for H2RA, 8 (9.4%) for misoprostol, 145 (9.6%) for rebamipide, and 389 (14.3%) for eupatilin. The multivariable analysis of each GPA combination showed that PPIs (HR, 0.30; 95% CI, 0.20-0.44), H2RAs (HR, 0.35; 95% CI 0.29-0.43), misoprostol (HR, 0.47; 95% CI, 0.23-0.95), and rebamipide (HR, 0.43; 95% CI, 0.35-0.51) significantly reduced the risk of occult GI bleeding. In contrast, concomitant use of eupatilin did not reduce the risk of occult GI bleeding compared to that among nonusers. Further, we evaluated the comparative effectiveness of the GPA co-treatments. Compared to that associated with PPI co-treatment, the H2RA, misoprostol, rebamipide, and eupatilin co-treatment–associated HRs for occult GI bleeding were 1.19 (95% CI, 0.79-1.79), 1.58 (95% CI, 0.72-3.46), 1.44 (95% CI, 0.96-2.16), and 3.25 (95% CI, 2.21-4.77), respectively (Table 3).

**Table 3:** The effect of GPAs on the risk of gastrointestinal bleeding in NSAID users.

	Multivariable analysis		
	HR	95% CI	*P* value
**Concomitant GPA use versus non-use**			
None	1.00	Reference	
PPI	0.30	0.20-0.44	<0.001
H2RA	0.35	0.29-0.43	<0.001
Misoprostol	0.47	0.23-0.95	0.035
Rebamipide	0.43	0.35-0.51	<0.001
Eupatilin	0.98	0.86-1.12	0.74
**GPA use versus PPI co-therapy**			
PPI	1.00	Reference	
H2RA	1.19	0.79-1.79	0.409
Misoprostol	1.58	0.72-3.46	0.257
Rebamipide	1.44	0.96-2.16	0.078
Eupatilin	3.25	2.21-4.77	<0.001

The analysis adjusted for sex, age, body mass index, creatinine level, and co-morbidities (hypertension, cerebrovascular disease, diabetes mellitus and ischemic heart disease).

To evaluate the consistency of the effect of GPAs on occult GI bleeding, we performed subgroup analyses of the factors affecting GI bleeding (Table 4). The prespecified subgroup analysis did not show heterogeneity of the risk of occult GI bleeding in GPA co-treatment or significant interactions with respect to age (<60 vs. ≥60 years), sex (male vs. female), renal function (creatinine < 1.2 mg/dL vs. creatinine ≥1.2 mg/dL), and BMI (<25 kg/m^2^ vs. ≥25 kg/m^2^).

**Table 4:** Risk of GI bleeding by drug combinations in clinically-relevant subgroups.

Subgroup	Drug combinations					
	NSAID monotherapy	PPI	H2RA	Misoprostol	Rebamipide	Eupatilin
**Age**						
<60 years	reference	0.17 (0.08-0.37)	0.41 (0.31-0.54)	0.85 (0.40-1.81)	0.48 (0.37-0.62)	0.87 (0.69-1.09)
≥60 years	reference	0.42 (0.27-0.66)	0.36 (0.28-0.48)	0.18 (0.02-1.25)	0.54 (0.41-0.71)	0.95 (0.81-1.12)
**Sex**						
Male	reference	0.32 (0.16-0.66)	0.49 (0.35-0.68)	0.45 (0.14-1.40)	0.53 (0.38-0.73)	1.29 (1.00-1.65)
Female	reference	0.29 (0.18-0.45)	0.31 (0.24-0.39)	0.60 (0.25-1.45)	0.39 (0.31-0.50)	0.86 (0.74-1.01)
**Renal function**						
Cr < 1.2 mg/dl	reference	0.22 (0.13-0.36)	0.32 (0.26-0.41)	0.52 (0.19-1.41)	0.37 (0.30-0.47)	0.92 (0.79-1.07)
Cr ≥ 1.2 mg/dl	reference	0.57 (0.32-1.04)	0.45 (0.32-0.63)	0.50 (0.19-1.36)	0.60 (0.43-0.84)	1.16 (0.88-1.53)
**Obesity**						
BMI < 25	reference	0.25 (0.11-0.57)	0.37 (0.25-0.53)	0.99 (0.31-3.12)	0.45 (0.31-0.65)	0.77 (0.56-1.05)
BMI ≥ 25	reference	0.09 (0.02-0.35)	0.27 (0.18-0.41)	0.39 (0.05-2.28)	0.30 (0.19-0.44)	0.86 (0.67-1.10)

Estimated from Cox proportional hazard models. Multivariable model was adjusted for age, sex, body mass index, creatinine level, and co-morbidities.

GI, gastrointestinal; NSAID, nonsteroidal anti-inflammatory drug; PPI, proton pump inhibitor; H2RA, histamine-2 receptor antagonist; Cr, creatinine; BMI, body mass index; IHD, ischemic heart disease.

## Discussion

In this large cohort study, we investigated whether various GPA co-treatments are effective in reducing the risk of GI bleeding in NSAID users. We found a 36% reduction in the risk of occult GI bleeding in GPA users compared to that in nonusers. Our sub-analysis showed that PPIs, H2RAs, misoprostol, and rebamipide significantly reduced the risk of GI injury. Our results also showed no significant difference in occult GI bleeding between those using GPAs except eupatilin users. Therefore, our findings indicate that GPA co-treatment with agents such as rebamipide and misoprostol, as well as acid suppressants, including PPIs and H2RAs, are effective in preventing GI injury in NSAID users; however, a small difference in efficacy cannot be excluded.

GI bleeding is a major adverse clinical outcome associated with the use of NSAIDs. Current evidence shows that NSAIDs increase the risk of lower GI bleeding to a similar extent as that in the upper GI^3–5^. In recent times, lower GI bleeding due to NSAID use has become an important issue because of the increased use of NSAIDs in our aging society, and older age is an important risk factor for lower GI bleeding^4,5,22^. Acid suppressants such as PPIs and H2RAs are typically associated with a decreased risk of upper GI bleeding^23^; however, they do not have protective effects against lower GI injury due to NSAIDs. Furthermore, some studies have reported that PPIs and H2RAs are associated with an increased risk of lower GI bleeding^24–27^. At present, effective means to prevent NSAID-associated lower GI injury are not available. However, misoprostol, rebamipide, antibiotics, and sulfasalazine have been shown to prevent NSAID-associated lower GI injury in animal models and in human studies with small sample size^11,12,21,28–31^.

The endpoint of a study concerning NSAID-associated GI injury is also important. The endpoints of previous studies were mostly limited to upper GI bleeding, such as gastroduodenal ulceration confirmed by endoscopy and overt GI bleeding events, namely melena or hematemesis. Furthermore, previous prospective randomized clinical trials have focused primarily on upper GI events^14,32–34^; however, recently, several studies have used the novel comprehensive composite endpoint of both upper and lower GI bleeding, defined as a clinically significant drop in the hemoglobin level (≥2 g/dL)^35,36^. The use of the absolute cut-off of 2 g/dL has been advocated by the US Food and Drug Administration and supported by the European Medicines Agency as well as other researchers. A hemoglobin drop of ≥2 g/dL and/or a hematocrit drop of ≥10% is reflective of clinically significant upper or lower GI bleeding and could prompt physicians to intervene with new clinical decisions or interventions. Based on these considerations, this practical endpoint has been used as an important outcome in a number of large GI outcome trials evaluating GI toxicity in NSAID users^14,32,33,36^. Compared to the estimation of overt GI events, the confirmation of a decrease in hemoglobin might be a better indicator of the impact of medications on the development of overall GI tract injury.

PPIs and H2RAs are well-known, effective GPAs for preventing upper GI bleeding in NSAID or low-dose aspirin users^7,37,38^. Despite the widespread use of PPIs, there remain concerns about several potential risks. Previous studies have reported that PPI use may be associated with an increased risk of several infectious diseases including *Clostridium difficile* infection, other enteric infections, pneumonia, and osteoporotic fractures, mainly through acid suppression in the stomach^39,40^. There also have been concerns about potential drug interactions^41^. Concomitant use of PPIs in patients receiving clopidogrel has been reported to increase the risk of coronary events^42^. Because of the anxiety about the long-term safety of PPIs, clinicians are looking for alternative GPAs. Indeed, in this study, GPAs including eupatilin, rebamipide, and misoprostol, which do not cause acid suppression, accounted for more than 60% of the total GPAs used. Eupatilin, the standardized extract of *Artemisia asiatica*, has been widely prescribed for the treatment or prevention of gastric mucosal lesions of acute and chronic gastritis in Asia. It has been shown to have potent anti-inflammatory and/or protective effects against GI lesions in both animal models and in humans^43–45^. However, our results revealed that eupatilin did not reduce the risk of GI bleeding in NSAID users. Further, our findings showed that rebamipide and misoprostol have similar efficacy in reducing the risk of GI bleeding in NSAID users compared to that of PPIs and H2RAs. Several studies have revealed that misoprostol not only prevents upper GI injury in NSAID users but is also effective against small and large bowel injury^12,31,46,47^. Rebamipide is a well-known mucoprotective drug against both NSAID-induced gastric mucosal injury and small-intestinal mucosal injury^21,48^. Our results showed no significant difference in overall GI bleeding among the GPAs.

Several limitations need to be considered in the interpretation of our results. First, it was performed at a single tertiary referral center. Second, this study had a retrospective observational design. However, prospective studies are difficult to perform for rare events such as GI bleeding, given the requirement for a large number of patients to be followed over a long period. Furthermore, outcomes in observational studies are attractive because they may be more representative of “real-world” practice compared to the results from the restricted populations in randomized trials. Third, because our study used a ≥2 g/dL drop in hemoglobin level as the definition of bleeding, we included people who underwent routine asymptomatic laboratory follow-ups. Patients who underwent routine asymptomatic laboratory follow-up may have had other comorbidities or symptoms. This may result in a bias. Fourth, although we controlled for several important confounding factors in the analysis, we cannot exclude the possibility of residual confounding due to unmeasured factors such as *Helicobacter pylori* status and smoking status, which are important risk factors for GI bleeding. Fifth, other possible causes for the decrease in hemoglobin, such as menorrhagia in female patients, were not excluded. In this study, the patients with hemoglobin drop were asymptomatic; therefore, the cases of hemoglobin drop other than occult GI bleeding might be very small. Finally, information on over-the-count medication was not available from the hospital database. Over-the-count NSAIDs and GPAs are usually for short-term use, but an underestimation of NSAID and GPA use in our study is conceivable.

In conclusion, we found that the risk of occult GI bleeding was higher in NSAID users without GPA co-treatment in this large cohort. Our findings also revealed that increasing age and a history of ischemic heart disease were significant risk factors for GI bleeding. The risk of GI bleeding was reduced by using concomitant GPAs. Among the GPAs, rebamipide and misoprostol, as well as acid suppressants such as PPIs and H2RAs, were effective in preventing GI bleeding. Rebamipide or misoprostol co-treatment could be recommended as a reasonable alternative in NSAID users.

## Methods

### Study population

This cohort study was performed using data extracted from the Clinical Data Warehouse (CDW) Darwin-C of Samsung Medical Center. We screened 12,871 consecutive patients with osteoarthritis or rheumatoid arthritis, aged 20 years or older who received NSAIDs for at least 1 month at the outpatient department of Samsung Medical Center, South Korea, from January 1995 to December 2014. Patients entered the cohort on the day they received their first NSAID prescription at this hospital. Patients were excluded if they met any of the following criteria: concomitant use of low-dose aspirin, use of antiplatelet agents other than aspirin, use of anticoagulants such as warfarin, dabigatran, rivaroxaban, apixaban, and edoxaban, or any steroid; use of two or more NSAIDs; and use of two or more GPAs. Furthermore, we excluded patients with a history of inflammatory bowel disease, chronic liver disease, GI surgery, GI polyposis syndrome, malignancy, peptic ulcer disease, infectious diseases requiring antibiotics, immune-mediated diseases or autoimmune diseases requiring immune-suppressants, or chronic kidney disease including stages 4 and 5. In addition, we excluded patients who underwent surgery or other medical procedures patients during the study period. Finally, 9,133 patients who received NSAIDs for at least 1 month and whose baseline and follow-up data for hemoglobin levels were available, were included in this study. This study was approved by the institutional review board of the Samsung Medical Center and conducted in accordance with the Declaration of Helsinki. The requirement for informed consent was waived since we used only de-identified data collected from the CDW.

### Data collection

The CDW of Samsung Medical Center includes the prescription date and drug name, quantity, and dosage. It also includes patient demographic information such as age and sex, anthropometric measurements, laboratory data, and diagnostics codes according to the International Classification of Diseases, 10^th^ revision. The following non-selective NSAIDs were included: aceclofenac, etodolac, fenoprofen, ibuprofen, indomethacin, ketorolac, loxoprofen, mefenamic acid, meloxicam, morniflumate, nabumetone, naproxen, pelubiprofen, piroxicam, sulindac, talniflumate, and zaltoprofen. GPAs included in this study were PPIs (esomeprazole, lansoprazole, omeprazole, pantoprazole, and rabeprazole), H2RAs (cimetidine, famotidine, and ranitidine), misoprostol, rebamipide (Mucosta®), and eupatilin (Stillen®). The exposure period began on the NSAID prescription date and ended at the end of the prescribed amount. The patients were divided into two main groups: NSAID without GPA and concomitant GPA use during NSAID treatment. The latter was further divided into five subgroups based on the different GPA co-treatments. The analysis was restricted to the first NSAID treatment with or without GPA co-treatment by censoring follow-up time after any treatment switch or withdrawal. Comorbidities identified from diagnostic codes were classified into four diseases including hypertension, diabetes mellitus, cerebrovascular disease, and ischemic heart disease.

### Outcome definition

The primary endpoint was occult GI bleeding, defined as a decrease of at least 2 g/dL from baseline in the hemoglobin level. The baseline hemoglobin level was defined as the value measured until one month before the NSAID start date. Laboratory examinations including determination of hemoglobin levels were performed every 1–3 months during the follow-up period. The patients were followed up for 12 months. Bleeding events that occurred during the NSAID treatment or within 30 days after the discontinuation of treatment were included in the analysis.

### Statistical analysis

Continuous variables are reported as means ± standard deviation values, while categorical variables are presented as percentages. Continuous variables were compared between the groups by using Student’s *t*-test, while categorical variables were compared using the Chi-squared test. Descriptive statistics were used to summarize the baseline characteristics of the patients according to the GPA use. The primary endpoint was the development of incidental GI bleeding. Patients were followed from the first day of NSAID treatment until the development of GI bleeding or the last hemoglobin examination among those who did not develop GI bleeding. Cumulative incidence rates were calculated using the Kaplan-Meier method and analyzed using the log-rank test. Cox regression models were used to estimate the adjusted hazard ratios (HRs) with 95% CIs for incidental GI bleeding, comparing concomitant use of GPA plus NSAID with NSAID use without GPA. To evaluate whether other GPAs, compared to PPIs, showed comparable efficacy for preventing GI bleeding, the adjusted HRs for each GPA were determined using the concomitant PPI users as the reference group. The multivariable model was adjusted for age (years), sex, BMI (kg/m^2^), creatinine level (mg/dL), and comorbidities.

Sensitivity analyses were conducted to evaluate the consistency of the effect of GPAs on occult GI bleeding according to the clinically-relevant groups, defined by age (<60 vs. ≥60 years), sex (male vs. female), renal function (creatinine < 1.2 mg/dL vs. creatinine ≥1.2 mg/dL), and BMI (<25 kg/m^2^ vs. ≥25 kg/m^2^). Statistical analyses were performed using R version 1.0.153 (RStudio, Inc.) and SPSS version 24.0 (IBM Corp.).
